# Supplementary material for: Identification of photoperiod-induced specific miRNAs in the adrenal glands of Sunite sheep (Ovis aries)
Source: Front Vet Sci. 2022 Jul 22;9:888207. doi: 10.3389/fvets.2022.888207 (PMC9354845; doi:10.3389/fvets.2022.888207)
Supplement: Supplementary file 1 [file Data_Sheet_1.ZIP › Supplementary file/miRDeep2 file.zip!/miRDeep2 file/Know_Structure/LP42A2/oar-mir-541.pdf]

miRBase precursor : oar-mir-541
Total read count : 106
oar-miR-541-5p read count : 99
oar-miR-541-3p read count : 7
remaining reads : 0

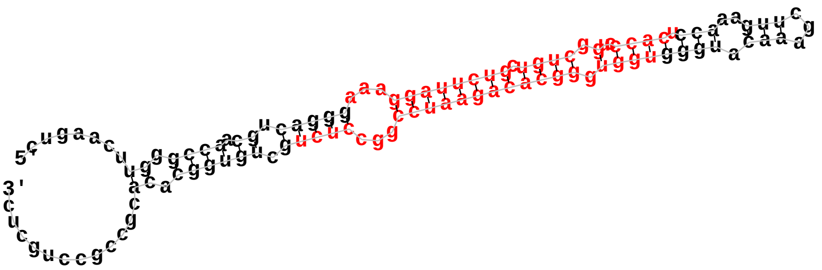

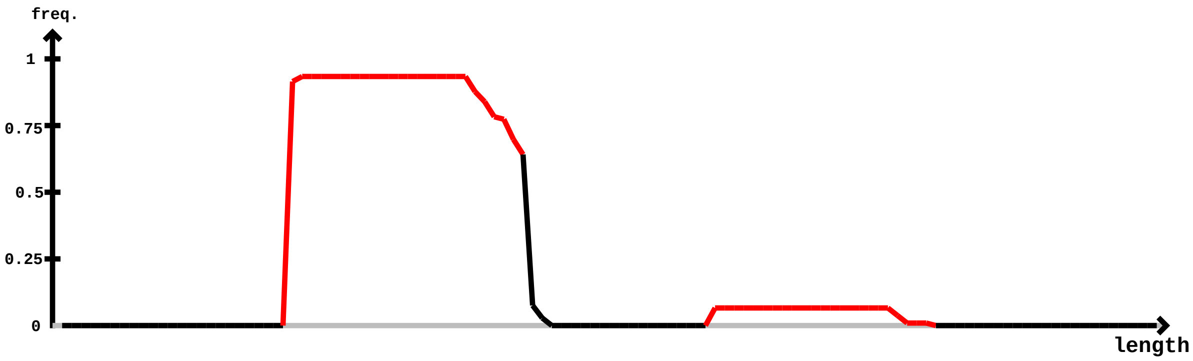

oar-miR-541-3p

oar-miR-541-5p

```
5'- cugaacuugggccaacgucagggaaaggauucugcugucggucccacuccaaaguucgaaacauggguggugggcacagaauccggccucugcuguggcacacgccgccugcuc  -3'  exp
    ........((((..(((.(((((...((((((((.((((.(..((((.(((..(((...))).)))))))).))))))))))))...))))).)))(((....)))))))....  reads  mm  sample
    .......................aaaggauucugcugucggu........................................................................  6  0  seq
    .......................aaaggauucugcugucgguc.......................................................................  4  0  seq
    .......................aaaggauucugcugucggucc......................................................................  6  0  seq
    .......................aaaggauucugcugucgguccc.....................................................................  1  0  seq
    .......................aaaggauucugcugucgguccca....................................................................  8  0  seq
    .......................aaaggauucugcugucggucccac...................................................................  6  0  seq
    .......................aaaggauucugcugucggucccacu..................................................................  58  0  seq
    .......................aaaggauucugcugucggucccacuc.................................................................  5  0  seq
    .......................aaaggauucugcugucggucccacucc................................................................  3  0  seq
    ........................aaggauucugcugucggucccacu..................................................................  2  0  seq
    ...................................................................uggugggcacagaauccgg............................  3  0  seq
    ...................................................................uggugggcacagaauccggc...........................  3  0  seq
    ...................................................................uggugggcacagaauccggccuc........................  1  0  seq
```
